# Emerging Role of Taste Receptors, Entero-Endocrine Cells in Type 2 Diabetes and Metabolic Disorders

**DOI:** 10.3390/nu18050759

**Published:** 2026-02-26

**Authors:** Kyaw Linn Su Khin, Sepideh Youssefi, Qian Yang, Amanda J. Page, Abdolrahman S. Nateri, Sally Eldeghaidy, Richard L. Young, Iskandar Idris

**Affiliations:** 1 Centre of Metabolism, Ageing & Physiology, School of Medicine, University of Nottingham, Royal Derby Hospital, Uttoxeter Road, Derby DE22 3DT, UK; kyawlinn.sukhin@nottingham.ac.uk; 2 Cancer Genetics & Stem Cell Group, The BioDiscovery Institute, University of Nottingham, Nottingham NG7 2RD, UK; sepideh.youssefi@nottingham.ac.uk (S.Y.); a.nateri@nottingham.ac.uk (A.S.N.); 3 Division of Food, Nutrition and Dietetics, School of Biosciences, Sutton Bonnington Campus, University of Nottingham, Sutton Bonington LE12 5RD, UK; qian.yang@nottingham.ac.uk (Q.Y.); richard.young@adelaide.edu.au (R.L.Y.); 4 Sensory Science Centre, Division of Food, Nutrition and Dietetics, Sutton Bonnington Campus, University of Nottingham, Sutton Bonington LE12 5RD, UK; 5 Lifelong Health, South Australia Health and Medical Research Institute (SAHMRI), Adelaide, SA 5000, Australia; amanda.page@adelaide.edu.au; 6 Intestinal Nutrient Sensing Group, Adelaide Medical School and Centre Translating Nutritional Science to Good Health, University of Adelaide, Adelaide, SA 5005, Australia; 7 Academic Unit of Translational Medical Sciences, School of Medicine, University of Nottingham, Nottingham NG7 2UH, UK; 8 Sir Peter Mansfield Imaging Centre, School of Physics and Astronomy, University of Nottingham, Nottingham NG7 2RD, UK; 9 National Institute of Health Research (NIHR), Nottingham Biomedical Research Centre (BRC), Derby Road, Nottingham NG7 2UH, UK

**Keywords:** taste receptors, gut hormones, diabetes, obesity, metabolism, enteroendocrine cells

## Abstract

Type 2 diabetes (T2D) is a major global healthcare challenge and burden on the quality of life in affected individuals. While lifestyle management is the mainstay treatment for T2D, the advent of gut-incretin-based therapies with powerful effects on metabolic health, appetite and weight regulation has focussed attention on the role of the gut in the risk, progression and management of T2D. Beyond the tongue, intestinal sweet taste receptors (STRs) are increasingly being identified and functionally characterised. Growing evidence now supports a role for nutrient-activated (e.g., sugars) intestinal STRs in the release of gut hormones from enteroendocrine cells (EECs) and the control of blood glucose and body weight. However, the specific STR pathway and mechanisms linking STRs to these homeostatic controls are poorly understood, with a notable gap existing between evidence from preclinical studies and clinical validation. This review explores intestinal STR-EEC functions and the evidence on how these functions regulate glucose metabolism and energy homeostasis. We further discuss the impact of environmental and dietary factors on these signalling pathways. Full knowledge of the signalling and regulation of intestinal STR-EEC and integrated neural pathways will bridge the current knowledge gap, with a high potential to develop new novel strategies targeting STRs or EECs that preserve hedonic taste rewards and reduce cravings, as well as improve the management of individuals with metabolic diseases.

## 1. Introduction

Type 2 diabetes (T2D) is a highly prevalent chronic disorder characterised by elevated blood sugar levels due to abnormal β-cell function and insulin action, risking long-term micro- and macro-vascular complications. The International Diabetes Forum Atlas reported 537 million adults worldwide living with diabetes in 2021, a number projected to rise to 783 million by 2045, with 90% of these classified as T2D [1].

Conventional treatment strategies for T2D include lifestyle interventions, oral hypoglycaemic medications, insulin therapy and, more recently, DPP4 inhibitors (dipeptidyl peptidase-4), SGLT2 (sodium-glucose co-transporter-2) inhibitors, and injectable and oral incretin-based therapies [2]. Despite their effectiveness in managing elevated blood glucose levels and reducing the risk of long-term complications, achieving optimal glucose levels continues to challenge many patients due to treatment side effects and non-adherence to prescribed lifestyle and therapeutic interventions [2]. Despite advancements in treatment, significant gaps remain in understanding the full biological mechanisms underlying T2D, which has spurred research into novel therapeutic targets that could offer more effective management. In this context, the regulation of eating behaviour by gastrointestinal (GI) enteroendocrine cells (EEC) and the gut–brain axis has emerged as a central theme of pharmaceutical and nutritional research.

Excessive consumption of energy-dense food and beverages in the modern diet is strongly linked to the development of obesity, T2D, cardiovascular disease and certain cancers [3]. The GI tract (GIT) serves as a major determinant of energy homeostasis due to the complex interaction of nutrient sensing by lingual and intestinal taste receptors, gut hormones secreted from EECs and the activation of the brain’s reward pathways. Increased understanding of the mechanisms governing these gut–brain pathways is therefore crucial for the development of novel strategies to manage obesity and T2D.

Historically, the study of taste receptors has focused on lingual nutrient signalling in the regulation of appetite and reward. However, recent preclinical and emerging clinical findings highlight the potential for post-oral taste receptors in the pathophysiology of T2D, notably intestinal taste receptors, which have the capacity to modulate gut hormone secretion from EECs and, therein, metabolic outcomes [3]. However, the precise mechanisms through which taste receptor-equipped EECs contribute to glucose homeostasis and the development of insulin resistance remain poorly understood. This review aims to bridge this gap by focusing on the genesis, regulation and functional implications of taste receptor-equipped EECs.

This review will first provide an overview of taste receptor structure, distribution and function. Next, we discuss the genesis of EECs and their capacity to detect luminal nutrients and respond via post-receptor signalling. We then explore how intestinal nutrient sensing is regulated in gut EECs and the consequences of this for gut hormone secretion. Finally, we highlight how the gut–brain axis is regulated and propose future research to develop therapeutic and nutritional avenues for effective treatments for T2D and related metabolic disorders.

## 2. Taste Receptors

### 2.1. Lingual Taste Receptors

**Figure 1:**
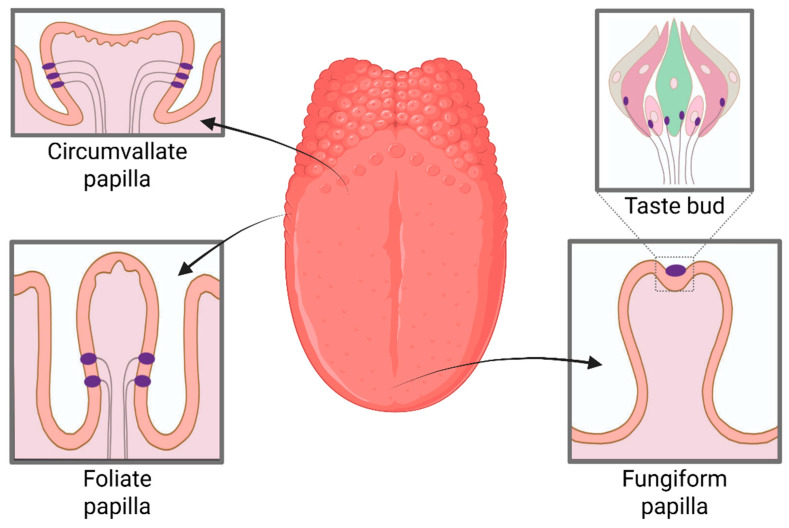
Illustration of lingual papillae types, demonstrating the location of each papillae on the tongue and its shape.

An average adult human tongue has 2000–10,000 taste buds, 75% of which are located on the dorsal aspect of the tongue, located in small elevations called papillae [4]. Of the four papillae types, fungiform, foliate, and circumvallate papillae harbour taste receptor cells (TRC) that sense the five basic tastes of sweet, bitter, sour, salty, and *umami* [5]. Fungiform papillae are mushroom-shaped structures, 87% of which are located at the anterior 2 cm surface of the tongue, and circumvallate papillae form an inverted V at the posterior of the tongue [5], whereas foliate papillae are small vertical folds located on the lateral sides of the tongue (location and shapes of the papillae are demonstrated in Figure 1) [5]. Filiform papillae are the most numerous lingual papillae, lacking TRCs but connecting to the trigeminal nerve to transduce touch, temperature, nociception, and texture [5].

Taste receptor cells (TRC) within lingual taste papillae are subdivided into three morphological subtypes (I-III), each of which responds uniquely to gustatory stimuli [6,7]. Type I TRCs have glial-like supportive roles and a role in the transduction of salt tastes, though the underlying mechanisms remain largely undefined [4,5]. Type II TRC express G protein-coupled receptors (GPCRs) tuned to detect sweet, *umami* and bitter taste [8,9] and are the focus of much research. Type II TRC express two main GPCR taste families: type 1, or T1R (T1R1, T1R2 or T1R3), and type 2, the T2R family. Heterodimeric T1R2 + T1R3 function as a broadly tuned STR for all sweet tastants, including hexose sugars and non-nutritive sweeteners (NNS) [10], while rarer homomeric T1R3 are more sensitive to hexose sugars than NNS [11]. In contrast, heterodimeric T1R1 and T1R3 function as L-glutamate (i.e., monosodium glutamate) responding to *umami* receptors, while the T2R family comprise over 25 bitter taste receptors (BTR) with individual members tuned to discrete bitter tastants [12]. The BTR, *TAS2R38,* is widely studied as an oral marker for differences in taste perception, food preferences and a potential link to body mass composition [13,14]. Presynaptic type III cells directly respond to sour stimuli [15]. Different tastants stimulate gustatory afferent nerve fibres located at the base of taste buds, and these sensory neurons carry the signals to the brain (the geniculate, petrosal, and nodose cranial ganglia) [15]. Emerging evidence also supports the existence of additional basic lingual tastes that detect fat, calcium, kokumi, metallic and ammonium chloride. Fat taste has been widely proposed in recent research as meeting the criteria for a basic taste, although its mechanisms and classifications are not yet universally defined [16,17,18].

**Figure 2:**
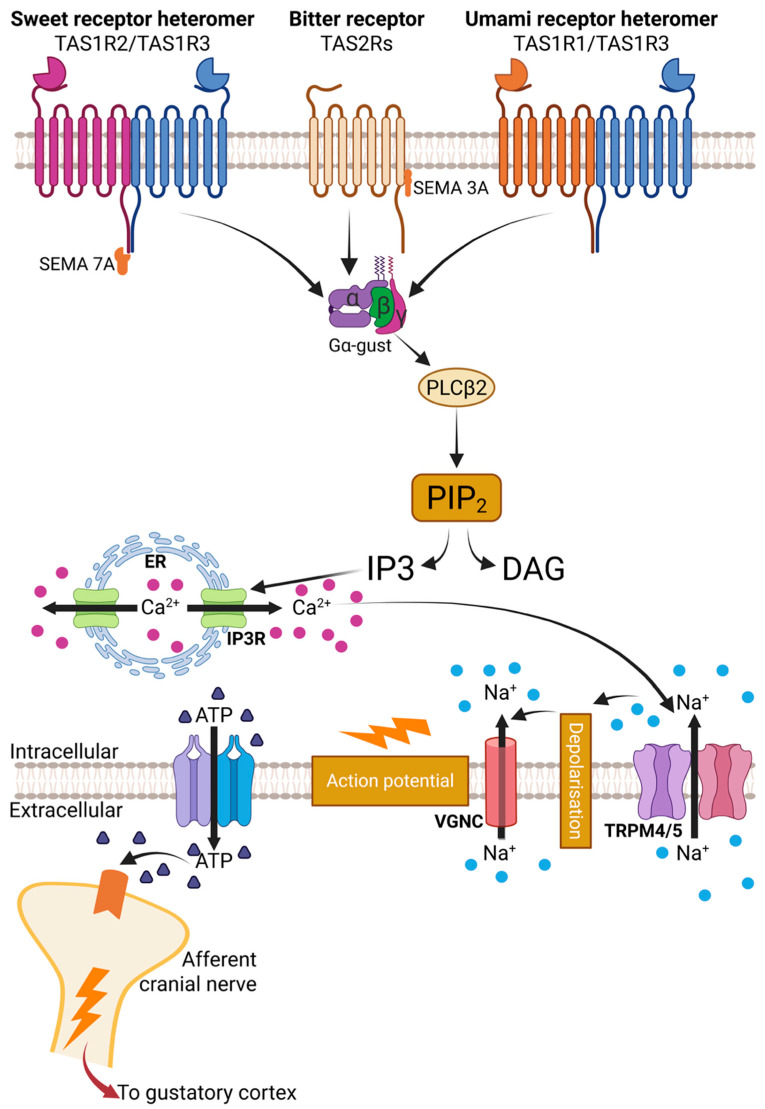
Signal transduction pathway for lingual taste GPCR [19]. Stimulation of T1R2/T1R3 (sweet taste receptors, STR), T2Rs (bitter receptors), and T1R1/T1R3 (*umami* taste receptors) on type II taste receptor cells (TRC) leads to activation of Gα-gustducin and Gα-q/11. This in turn releases inositol 1,4,5-triphosphate (IP3) and diacylglycerol (DAG) by activation of phospholipase C isoform β-2 (PLCβ-2). IP3 activates IP3 receptors and releases intracellular calcium (Ca^2+^) from the endoplasmic reticulum (ER). This, in turn, activates the transient receptor potential cation channel subfamily M member 5 (TRPM5), leading to depolarisation and subsequent activation of voltage-gated sodium channels (VGNC). This then activates calcium homeostasis modulator 1 and 3 (CALHM1/3), resulting in the release of adenosine triphosphate (ATP). Increased ATP subsequently activates ionotropic purinergic receptor 2X_2_ and 2X_3_ (P2X2/3) channel synapses on afferent cranial nerves to relay these signals to the gustatory cortex for sensory perception.

Taste receptors are coupled to taste-specific G-proteins comprising alpha, beta and gamma subunits (Figure 2). Gα-gustducin and Gα-transducins convey bitter and sweet signals, while Gγ13 is required for bitter taste perception through Gα-gustducin [19] (Figure 2). Binding of different nutrients to lingual taste receptors triggers activation of Gα-gustducin, which initiates a cascade involving the activation of phospholipase C-β2 (PLCβ2), release of intracellular calcium from inositol-1-4,5-triophosphate (IP3) stores [19], opening of TRPM5 (transient receptor potential channel M5) channels and activation of voltage-gated sodium channels (VGNC), leading to membrane depolarization and adenosine triphosphate (ATP) release [19,20] to activate presynaptic cells (type III TRC) (Figure 2). Type II TRCs, in turn, activate afferent sensory fibres of the chorda tympani and glossopharyngeal nerve via serotonin (5-HT) or noradrenaline (NA) signals, which transmit these sensory signals to the insular cortex in the brain [19,20] (Figure 2).

### 2.2. Intestinal Taste Receptors

Post-oral taste receptors are widely expressed in the intestine, nasal and lung epithelium, pancreas, brain, heart, kidney and bladder, and are well-recognised in both human and animal studies [21]. Intestinal taste receptors serve a critical role as they interface between the wide range of nutrient cues associated with ingested food and the physiological outcomes within and beyond the GIT [22]. Like lingual TRC, subsets of intestinal EECs possess microvilli exposed to ingesta [22] and are equipped with apical taste receptors similar to the tongue cued to detect glucose and other macronutrients, including non-nutritive compounds [23].

EECs lining the GIT are equipped with STRs and BTRs, with peak expression in the small intestine in both rodents and humans [23,24,25,26]. These intestinal EECs function analogously to lingual TRCs, where detection of taste cues triggers the release of gut hormones, including glucagon-like peptide-1 (GLP-1), glucagon-like peptide-2 (GLP-2) and peptide YY (PYY, peptide tyrosine tyrosine) from L-cells, cholecystokinin (CCK) from I-cells, and the hunger hormone ghrelin from P/D1 cells [21,27,28] (see Section 3 below). Like lingual TRCs, multiple components of the sweet taste pathway are expressed in individual intestinal taste cells, evidenced by the co-localisation of TIR2 and TIR3 proteins in rat jejunal epithelial cells [29]. Molecular studies have also revealed co-expression of T1R2, T1R3, Gα-gustducin and TRPM5 in subsets of glucose-dependent insulinotropic polypeptide- (GIP), 5-hydroxytryptamine (5-HT, serotonin)- and GLP-1-equipped EECs throughout the upper GIT in humans, with peak expression in the proximal small intestine [22,30,31]. Indeed, sweet tastants have been extensively linked to the gut release of key metabolic hormones that control the rate of gastric emptying, nutrient catabolism and absorption, insulin secretion, blood glucose and satiety [32,33].

T2Rs are expressed in small and large intestine-derived enteroendocrine L-cell lines, although the T2R-specific signal transduction pathways linked to gut hormone secretion here are not fully mapped [21,27,34,35]. Growing evidence supports a role for BTR signalling in the regulation of GLP-1 release [36]. Like STRs, BTRs are widely expressed in EEC subsets and have been shown to contribute to the regulation of gut hormone secretion. This is exemplified by T2R9-dependent GLP-1 release in rodents, where the BTR agonist, denatonium benzoate, increased GLP-1 secretion in a manner dependent on Gα-gustducin activation, reduced intracellular cAMP, and increased phospholipase activity and intracellular calcium [37,38]. Similarly, the bitter tastant quinine (a BTR agonist) was shown to variably control the rate of gastric emptying, but to augment plasma GLP-1 and therein, suppress energy intake in humans in a duodenal BTR-dependent manner [39,40,41,42,43,44,45]. Similarly, intraduodenal administration of quinine (300 or 600 mg) to individuals with T2D was shown to improve glycaemic responses to a carbohydrate-containing drink in a randomised double-blinded study, with 600 mg of quinine also leading to a modest increase in plasma GLP-1, C-peptide and glucagon [45]. Thus, bitter compounds could increase satiety by regulating GI motility and increasing the secretion of anorexigenic hormones like GLP-1, PYY, and CCK.

BTR activation has also been shown to modulate intestinal release of ghrelin and PYY to regulate appetite and energy balance, as well as play key roles in gut motility and gastric emptying, which govern nutrient absorption and metabolism [36,46]. The potential role of BTR signalling in ghrelin regulation has also been evaluated in mice, where gavage of the BTR agonists phenylthiocarbamide (PTC), denatonium benzoate or 6-n-propylthiouracil (PROP) increased total plasma ghrelin levels, while the BTR agonist quinine did not, indicating BTR specificity [46]. Ghrelin-releasing BTR agonists also increased food intake for 30 min after the gavage, followed by a prolonged decrease in food intake over the ensuing 4 h in mice replete, but not deplete, in Gα-gustducin. The subsequent decrease in food intake correlated with an inhibition of gastric emptying and involved a direct inhibitory effect of the BTR agonist on gastric contractility. Oral administration of PROP has also been shown to delay gastric emptying in mice [46]. Based on these animal and pre-clinical studies, intestinal EEC equipped with STRs and BTRs were proven to play a role in regulating gut hormone production, which controls gastric emptying, gut motility, appetite and energy regulation. The rise in ghrelin was triggered by bitter tastants, which appear to be reciprocal to appetite regulation due to an acute increase in hunger, and has downstream effects on delaying gastric emptying and potentially modulating other satiety hormones, which is favourable for obesity management, although results in human studies have been inconsistent.

## 3. Enteroendocrine Cells (EECs)

EECs are distributed throughout the GI mucosa and equipped to release gut hormones that regulate gut motility, insulin and food intake in response to meal-related stimuli. They have been classified traditionally based on the major hormone each produces and the location of peak cell density, exemplified by gastric D-cells (somatostatin), G-cells (gastrin), enterochromaffin-(EC)-cells (5HT), enterochromaffin-like cells (ECL)-cells (histamine) and P/D1-cells (ghrelin), proximal small intestine K-cells (GIP), I-cells (CCK), S-cells (secretin (SCT)), M-cells (motilin, ghrelin and somatostatin) and distal small intestine N-cells (neurotensin), and L-cells (GLP-1, GLP-2, PYY, insulin-like peptide 5 (INS5)) [10,47,48]. However, more recently, considerable overlap in hormone expression was noted in genetic studies among individual EECs [11] with L-cells having high transcript expression of GLP and PYY but also of GIP, CCK, SCT and neurotensin [49]. Similarly, K-cells showed transcription of GLP and CCK in addition to GIP, while I-cells expressed mRNA for GIP, GLP, SCT, NTS, and CCK [10,50,51]. This multi-hormonal expression challenges the accuracy of the conventional classification of EEC types.

In humans, the incretin hormones GLP-1 and GIP stimulate glucose-dependent insulin secretion, as well as somatostatin release [2]. While GIP receptors are localised to α-cells of the endocrine pancreas [52], GIP and GLP-1 exert broader metabolic effects on liver and muscle metabolism through their modulation of circulating insulin and glucagon levels [52]. These incretins exhibit opposing actions on glucagon, wherein GLP-1 suppresses, and GIP promotes glucagon release in a glucose-dependent manner [52]. Like GLP-1, oxyntomodulin also enhances glucose homeostasis by promoting glucose-dependent insulin secretion [53]. Furthermore, CCK contributes to glucose homeostasis by slowing gastric emptying, acting to reduce postprandial hyperglycaemia [54]. In contrast, animal studies have shown that intravenous administration of PYY inhibits glucose-stimulated insulin secretion [55]; however, the clinical significance of this is uncertain since this effect is not reported in humans [56].

### 3.1. Differentiation of EEC

The gut epithelium undergoes complete cell renewal every 3–5 days, rapidly adapting to its environment [57,58]. EECs originate from precursor neurogenin3 (*NEUROG3*)-positive cells that transiently express *NEUROG3* in a subset of newly formed intestinal epithelial cells, now destined to differentiate into EECs [59,60]. These cells then produce transcription factors neuronal differentiation 1 (*NEUROD1*), insulin gene enhancer protein *(ISL1*), pancreatic and duodenal homeobox 1 (*PDX1*), NK6 homeobox 1 (*NKX6-1*), and NK2 homeobox 2-2 (*NKX2-2*) [59,60]. Since these factors are common for differentiation in pancreatic islet cells, their co-expression highlights the common endodermal developmental origin of pancreatic and intestinal EECs [61]. The crucial role of *NEUROG3* in EECs development is firmly established, as *NEUROG3*-deficient mice exhibit a complete absence of intestinal EECs [10]. However, this dependence is region-specific, as gastric EECs are only partially reduced by *NEUROG3*-deficiency [10]; indeed, EECs in the gastric corpus likely originate from a hematopoietic lineage, while those in the gastric antrum are likely to be derived from extra-epithelial stem cells [59,62].

Notch signalling and expression of *MATH1* (mouse atonal homologue 1) directs intestinal progenitor cells toward the secretory lineage as goblet, Paneth and EEC [63,64]. Forkhead Box A1 and A2 (*FOXA1/FOXA2*) and *NEUROD1* act downstream of *NEUROG3* to drive differentiation into D and L-cell phenotypes or I and S cell phenotypes, respectively [65]. Transcription factors such as paired box 4 (*PAX4*), paired box 6 (*PAX6*), FLYWCH-Type Zinc Finger 1 (*FLYWCH1*) and *NEUROD1* regulate the transcription of hormone genes in EECs [58,66,67], while *NEUROD1* and *ARX* (Aristaless-related homeobox gene) have been identified as critical in vivo regulators of EEC differentiation [66,68]. Transcription factors associated with EEC differentiation are categorised into early-common (e.g., *NEUROG3*, *SOX4* (SRY-related HMG-box 4), *PAX4*, *ARX*, *FLYWCH1*) and middle-to-late (e.g., *PAX6*, *FOXA1*, *TRIM3* (tripartite motif containing 3)) categories [67,69].

The development and function of EEC, particularly the GLP-1-producing lineage, are significantly impaired in individuals with T2D. Decreased jejunal expression of early EEC transcription factors *RPS3, NEUROG3, PAX4*, and *SOX4* is noted in T2D, while late-stage factors like FOXA1 and TRIM3 are reported as upregulated [58,65,70]. *NKX2.2* is a key transcription factor in EEC lineage development [71], while *ARX*, *FOXA1*, *FOXA2*, *ISL-1*, and *PAX6* are essential for GLP-1 lineage specification [65,72,73,74,75]. Reduced jejunal expression of *SOX4* and *RUNX1T1* (Runt-related transcription factor 1) is also observed in T2D alongside elevated TRIM35, a transcription factor linked to the GLP-1 lineage [58]. Moreover, reduced proglucagon processing in the jejunal epithelial cells of individuals with T2D has been reported [58]. Collectively, these data add support that T2D and obesity are associated with reduced GLP-1 cell differentiation and maturation, resulting in reduced GLP-1 cell density in metabolic disease states.

### 3.2. Effects of Diet and Metabolic Status on Lingual and Intestinal Taste Receptor Functions

A variety of factors influence food choices and preferences, some of which begin in infancy, where the flavour of maternal food is transmitted to breast milk during feeding [76]. There is a large individual variation in oral sweet sensitivity, underscoring inter-individual differences in sugar consumption. Although the relationship between sweet taste preference and obesity risk remains inconsistent [77], increased sugar consumption in individuals accords with reduced sweet taste sensitivity [78]. Indeed, sucrose taste sensitivity is increased after a 12-week, calorie-restricted weight loss programme in females with obesity [79].

The association in taste changes in people living with type 1 and 2 diabetes has been studied using taste detection thresholds, taste recognition thresholds or suprathresholds. Reduced taste sensitivity was consistently found in T2D in more recent studies, particularly in those with hyperglycaemia or poorly controlled diabetes [80,81,82,83,84], although older studies showed no change in taste acuity [85,86,87]. In line with this, reduced taste sensitivity was found in patients with T1D [88,89], particularly with longer duration of diabetes (mean duration 4–20 years) [89,90,91]. This could be explained by the diabetes-associated asymptomatic microvascular complications, such as diabetic autonomic neuropathy, which is proven to have an impact on taste perception [83] or nephropathy, although these studies excluded patients who were diagnosed with diabetes complications. With longer duration of diabetes, it is common to have associated diabetic nephropathy, which can sometimes be asymptomatic [92]. Renal failure is a known factor influencing taste sensitivity [93]. However, there is no study to date testing for taste sensitivity in patients with diabetic nephropathy.

Additionally, individuals who have undergone Roux en-Y gastric bypass surgery show increased sweet taste sensitivity and, therefore, consume fewer high-carbohydrate-containing foods, which facilitates the maintenance of weight loss [82].

Genetic factors substantially influence individual differences in sweet taste sensitivity. Indeed, a study of 160 unrelated individuals found a strong link between variations in sucrose taste sensitivity and genetic variations in the *GNAT3* (gustducin alpha-3) gene, accounting for 13% of the observed differences in sweet taste perception [83,94]. Furthermore, multiple loss-of-function polymorphisms in the TAS1R family (i.e., TAS1R1, TAS1R2 and TAS1R3) have been identified, which influence sweet sensitivity in humans [84].

Sweet taste differences often correlate with sensitivity to other taste qualities, such as bitter [85]. The BTR *TAS2R38* mediates the lingual taste of glucosinolates PTC and PROP [95]. Polymorphisms in *TAS2R38* influence broad dietary selection, encompassing bitter produce, sweets, fats, and alcohol, and are directly linked to higher mean BMI in bitter non-tasters compared to super-tasters [95,96].

Prevailing and/or interventional diet patterns significantly impact EEC populations. For example, a high-fat, high-sugar diet in mice accelerated the differentiation of intestinal stem cells and also induced changes in enterocyte gene expression and function [97]. This led to an increased number of proximal enterocytes, which may in turn increase carbohydrate and fat absorption [97]. Interestingly, in a study that recruited morbidly obese patients undergoing bariatric surgery, the group with habitual high-fat low-carbohydrate diet consumption (>30% of fat and <50% of carbohydrate for total daily energy intake) was found to have higher GLP1-positive cells in the jejunum compared to a low-fat group [98]. The same study also analysed mice that were given an 8–14 weeks of a lipid-rich, low-carb diet (HFD) and demonstrated an increase in L-cell density by 30% compared to a control diet (CD) [98]. After 2–8 weeks, the HFD mice had impaired oral glucose tolerance test and fasting hyperglycaemia. Hormonal measurement of plasma GLP-1 revealed that HFD mice showed higher GLP-1 levels, which contributed to postprandial hyperinsulinaemia, but no inhibitory effect on glucagon after glucose bolus [98]. Thus, these data suggest that an obesogenic diet appears to induce functional maladaptation, which is thought to occur at the level of intestinal stem cells. Discrepancies between findings, however, exist, which may be due to factors such as selected mouse models (i.e., diet vs. genetically induced diabetes), type of diet (e.g., high-fat vs. high-fat, high-sugar diet), fat source and duration of dietary intervention, all of which can influence intestinal remodelling and response.

GIP mRNA levels were increased in purified K-cells from HFD-fed mice, despite no change in K-cell density [99]. Furthermore, a high-fibre diet was shown to increase colonic L-cell density in obese, leptin-deficient mice, and culture supplementation with short-chain fatty acids increased L-cell number in intestinal organoids [100,101]. While findings remain equivocal, these studies add support for the idea that maintaining a diet low in carbohydrates, high in fibre and with moderate fat safeguards EEC populations and their signalling, contributing to effective appetite and satiety control. However, given that these rodent studies assessed endpoints after 8–12 weeks, variation in the macronutrient compositions used in these studies and the lack of prospective human studies, it is challenging to define specific effects of diet from those concurrent with weight changes.

The exact mechanism by which diet regulates STR-equipped EEC function and distribution is currently unclear; however, jejunal STR expression and distribution are known to be dynamically regulated by luminal glucose exposure. For instance, high glucose concentrations rapidly traffic T1R2, T1R3, and Gα-gustducin proteins away from the jejunal brush border in humans. Concurrently, T1R2 transcript levels decrease in glucose-perfused jejunal loops, without altering blood glucose or other taste molecule transcripts [22].

Individuals with obesity and/or T2D exhibit profound alterations in EEC physiology. Specifically, they show altered intestinal release of key hormones (GLP-1, GIP, CCK, PYY) [65], concurrent with the reduced EEC differentiation and hormone synthesis associated with obesity [65]. A decrease in both taste bud density [102,103] and jejunal L-cell populations is also observed in obesity, independent of T2D status [58]. Although RYGB surgery increases postprandial GLP-1 and PYY release [104,105], this is largely attributed to rapid nutrient delivery to the distal small intestine [106] and potentially increased EEC number [107], rather than clarified effects on EEC density. The mechanisms driving these overall EEC alterations remain to be fully elucidated. Nonetheless, taken together, current evidence suggests that dietary intake, disease states such as diabetes and its relation to microvascular complications, macronutrient ingestions, and genetic factors could play important roles in regulating taste sensitivity, which in turn could affect food choices and preferences. While much of this data is supported by human studies, additional discrepancies in animal data exist that need to be correlated and translated with human studies.

## 4. Gut Nutrient Sensing and Gut Hormone Production

The receptors and signalling involved in nutrient sensing are complex; however, new insights into nutrient and taste receptor signalling pathways in intestinal EECs hold the potential to more effectively prevent and manage T2D.

### 4.1. Carbohydrate Sensing

Apical sodium–glucose co-transporter-1 (SGLT-1) is the primary intestinal glucose transporter in both humans and animals. SGLT-1 enables glucose absorption by co-transporting sodium along the electrochemical gradient established by the basolateral sodium-potassium ATPase [3]. Glucose then effluxes from enterocytes to the systemic circulation via basolateral-located facilitative glucose transporter 2 (GLUT2, a high-capacity, low-affinity bi-directional transporter) [22,108]. While intestinal STRs have been shown to regulate the expression and function of SGLT-1, less is known of the mechanisms involved in GLUT2 regulation [109].

Intestinal STR senses glucose and sweet tastants to activate Gα-gustducin signal transduction. Other glucose-sensing pathways are also present in the EEC. The ATP-sensitive K_ATP_ channel system, which functions as a metabolism-dependent ion channel, is a glucose sensor in a number of tissues, critically in pancreatic beta-cells [110]. K_ATP_ channels and glucokinase are expressed in both intestinal L-cells and K-cells in mice and humans, suggesting a potential role in peripheral glucose sensing [111,112]. However, the importance of K_ATP_ channels in intestinal glucose sensing remains unclear. This is because neither hyperglycaemia, per se, nor sulfonylurea drugs, which stimulate insulin secretion by closing K_ATP_ channels, trigger GLP-1 or GIP secretion [113]. This evidence strongly suggests that the K_ATP_ mechanism is not the primary or essential pathway for glucose-stimulated incretin release.

Transport of SGLT-1 substrates (e.g., glucose and galactose), however, is critical for GLP-1, GLP-2 and GIP hormone secretion [32] in L-cells that express *TAS1R* (TIR2) in both animal and human studies. The expression and activity of intestinal SGLT-1 have been shown to be enhanced in individuals with T2D, independent of the prevailing intake of dietary carbohydrates, blood glucose or insulin concentration changes [114], which is proposed to be due to altered mechanisms and signalling pathways involved in the regulation of SGLT-1 activity and expression.

In animals, studies in mice have demonstrated that the secretion of GLP-2 enhances the half-life of SGLT-1 mRNA in neighbouring absorptive enterocytes, leading to increased activity and expression of SGLT-1 [115]. Furthermore, sweet stimuli, including NNS, have the capacity to upregulate SGLT-1 expression and function, suggesting that STRs have the capacity to stimulate incretin hormone secretion by increasing SGLT-1 function [116]. A recent animal study, combining in vivo and in vitro approaches, showed that luminal sweet sensing via STRs and EEC-derived GLP-1 were important components in the regulatory pathway controlling GLUT2 expression [117]. However, evidence in humans remains unclear [116].

Interestingly, although intestinal STRs are expressed at similar levels in the duodenum of individuals with and without T2D, T1R2 expression decreased following the administration of enteral glucose in individuals without T2D but remained increased in individuals with T2D during hyperglycaemia, where this was linked to augmented glucose absorption [31]. The corollary is that intestinal STR dysregulation in T2D has the potential to further augment glucose absorption, and more chronically, exacerbate postprandial glycaemic excursions to worsen glycaemic control [3]. Further understanding of these mechanisms is critical to optimal management of T2D.

### 4.2. Fatty Acid Sensing

Intestinal EEC subsets sense lipids to initiate a signalling cascade that triggers CCK release (and subsequent gallbladder contraction), secretin release (leading to pancreatic enzyme secretion) and GIP and GLP-1 release [10]. Although multiple intestinal fatty acid receptors exist, those responsible for sensing long-chain fatty acids (LCFAs) are the most critical due to their essential role in inducing satiety signalling [10]. The principal GPCRs involved are FFAR4 (free fatty acid receptor 4), FFAR1 (free fatty acid receptor 1), GPR119 (G-protein coupled receptor 119), and the multi-functional transporter CD36. FFAR4, present on I-, K- and L-cells, primarily detects LCFA Gαq activation [10,114,115,118,119,120,121,122,123,124,125,126,127] and while its importance in energy regulation is established in preclinical settings [110], its role in humans remains unclear [128]. FFAR1 shares a similar EEC expression pattern to FFA4R, making their respective contributions to fatty acid sensing difficult to distinguish. Nonetheless, an FFAR1 agonist has been developed as a therapeutic for T2D/obesity due to its effects on fat-mediated insulin release [129]. Fasiglifam, an FFAR1 agonist, improved glycaemic control and reduced HbA1c with reduced risk of hypoglycaemia. However, this was withdrawn in a phase 3 trial due to its hepatotoxicity [129].

Combined FFAR1/4 agonists (icosabutate) have been proposed to confer greater anti-diabetic efficacy and have completed a phase IIb clinical trial for use in MASH (Metabolic dysfunction associated steatohepatitis), showing improvement of surrogate histological endpoints and biomarkers of liver injury, although there was no resolution of MASH [130]. Additionally, GPR119, activated by monoacylglycerols (triglyceride metabolic products), triggers GLP-1 and GIP release to augment insulin. However, this GPR119 pathway has not demonstrated sustained metabolic benefits in individuals with T2D [131,132].

### 4.3. Protein Sensing

Amino acids and oligopeptides are detected in intestinal EECs’ key receptors, including CaSRs (calcium-sensing receptors), GPRC6A (G-protein coupled receptor family C group 6 subtype A), the *umami* receptor (T1R1/R3) and the metabotropic glutamate receptor (mGluR) in intestinal EEC [10]. The CaSR is an abundant and versatile receptor in both human and rodent intestinal epithelial cells, which detects calcium and specific L-amino acids that are aromatic, aliphatic and polar [133]. CaSRs trigger amino acid-dependent release from gastrin-secreting G-cells, somatostatin-secreting D-cells, and CCK-secreting I-cells [133]. GPRC6A is also widely expressed as an amino acid sensor in human and rodent intestinal tissues [133] and may mediate GLP-1 release in response to L-ornithine and L-arginine, although this role requires further research [134,135]. The T1R1/R3 heterodimer functions as a *umami* sensor for L-amino acids like monosodium glutamate in the lingual epithelium in humans, whereas they respond to 20 standard amino acids in rodents, and are a major component of amino-acid-induced CCK release in intestinal secretin tumour (STC-1) cell lines and native mouse intestinal tissue [133,136]. Finally, metabotropic glutamate receptors (mGluRs), primarily known for modulating vasovagal reflexes (such as swallowing, gastric accommodation and emesis) in the CNS [137], are also located in the GIT of rodents and humans, where activation of mGluR5 triggers the release of PYY and neuropeptide Y (NPY), which influence appetite and eating behaviour [138].

## 5. Control of Gut Hormone Secretion in Humans and Their Physiological Actions

During fasting, a rise in the major orexigenic hormone ghrelin stimulates food intake, while increased somatostatin directly suppresses gastric acid secretion by targeting parietal (oxyntic) cells and indirectly limiting gastrin and histamine release, thereby reducing gastric digestive activity [10,139]. Following a meal, the early postprandial phase sees the sustained secretion of GIP from K-cells in the proximal small intestine, with its release critically dependent on sugar absorption via intestinal SGLT-1 [118]. Concurrently, the anorexigenic hormones GLP-1 and PYY are released during the postprandial phase, mainly from L-cells in the distal ileum, in response to nutrient cues, and much later, GLP-1 from colonic L-cells, in response to liberated short-chain fatty acids (SCFAs) [140]. The summary of principal gut hormones, their site of production and functions are summarised in Table 1.

Dietary components reaching the ileum trigger the release of these gut peptides, along with OXM, to activate the ‘ileal brake’ [140], a negative feedback loop that slows proximal GIT motility to optimise digestion, enhance nutrient uptake and promote satiation [140]. Furthermore, intestinal STRs play a direct or indirect role in mediating the release of GIP and GLP-1 via control of SGLT-1 activity [22]. This gut-hormone regulation is influenced by the circadian clock, which aligns GLP-1 secretion with feeding times [141]; indeed, studies in rats have demonstrated that disrupting a 12 h feeding cycle inverts the rhythm of GLP-1 and insulin secretion, highlighting an autonomous peripheral clock within intestinal L-cells [142].

The secretion of GLP-1 is also altered by circulating bile acids, gut microbiota, and circadian rhythm [37]. Microbial metabolites, such as SCFAs, resulting from complex carbohydrate fermentation, stimulate colonic GLP-1 release by activating FFARs. Conversely, gut microbe-deconjugated bile acids enhance GLP-1 secretion via activation of the bile acid receptor TGR5 (Takeda G protein-coupled receptor 5, also known as GPBAR1 or GPR131) [143,144].

## 6. Brain Responses to Nutrient Sensing: The Gut–Brain Axis

### 6.1. Brain Centres

**Figure 3:**
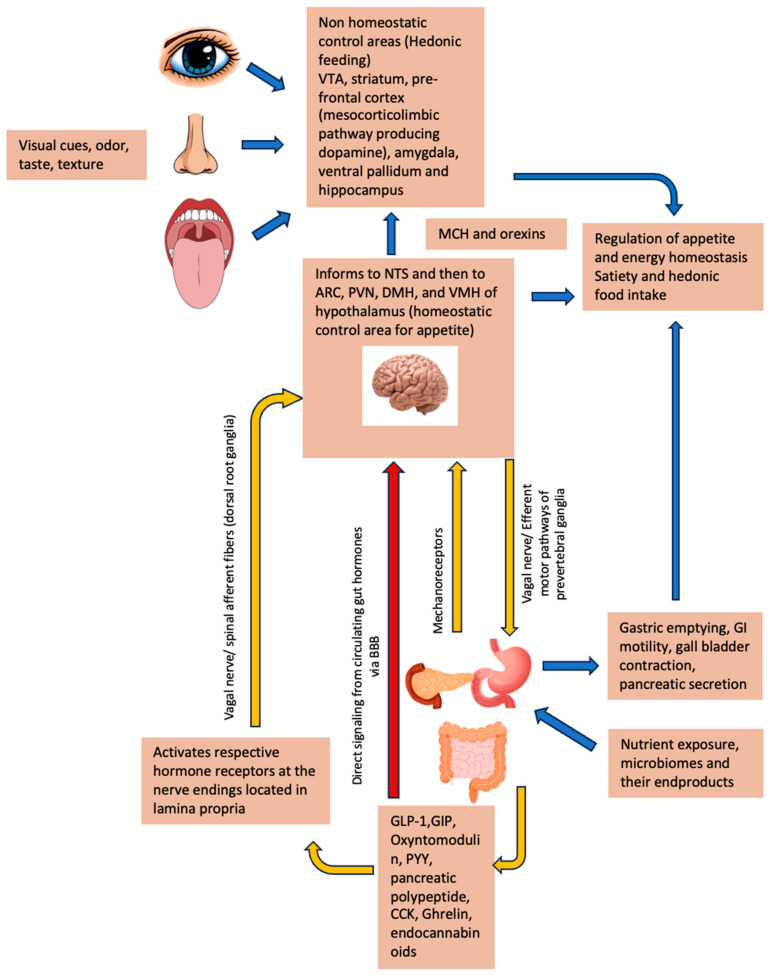
Gut–brain axis modulation of the hedonic system: This schematic demonstrates the communication between gut-derived hormones and their stimulation of the brain hedonic system. Abbreviations: ARC (arcuate nucleus), CCK (cholecystokinin), GIP (glucose-dependent insulinotropic polypeptide), GLP1 (glucagon-like peptide), PYY (Peptide YY), DMH (dorsomedial nucleus of hypothalamus), MCH (melanin-concentrating hormone), PVN (periventricular nucleus), VMH (ventromedial nucleus of hypothalamus), and VTA (ventral tegmental area).

Nutritional signals from the GIT are primarily transmitted via vagal afferents to the nucleus tractus solitarius (NTS), a crucial relay centre located in the dorsomedial medulla oblongata, lateral to the nucleus of the vagus nerve [145,146]. Although spinal afferents may also contribute [147], the NTS integrates this visceral information and relays it to higher brain centres, chiefly the hypothalamus, which is a central regulator of energy homeostasis [146]. Key hypothalamic nuclei involved include the arcuate nucleus (ARC), paraventricular, ventromedial, and dorsomedial nuclei, and the lateral hypothalamus [148] (Figure 3). Within the ARC, two antagonistic neuronal populations, the anorexigenic pro-opiomelanocortin/cocaine and amphetamine-regulated transcript (POMC/CART) neurons and the orexigenic neuropeptide Y/Agouti-related peptide (AgRP) neurons, integrate these signals to determine nutrient availability and energy balance [145,146]. In parallel, the area postrema (AP), a circumventricular organ located adjacent to the NTS featuring a fenestrated blood–brain barrier, provides a direct entry point for circulating gut hormones to access the central nervous system and influence these same hypothalamic centres [149] (Figure 3).

### 6.2. Gut Hormones and Their Interaction with Brain Centres

Gut hormones transfer signals centrally to the hypothalamic circuits to regulate energy homeostasis. The ARC houses receptors for these signals, including the GLP-1 receptor, Y2, Y4, and CCK-1 and 2 receptors. Circulating GLP-1 limits energy intake by slowing gastric emptying and directly stimulating satiety centres, notably the ARC, NTS and AP [145,150]. GLP-1 is also produced centrally by neurons in the caudal brainstem, a key site for integrating vagally mediated gut–brain signalling [145,150]. Similarly, CCK exerts peripheral effects primarily through vagal afferent fibres and paracrine mechanisms within the gut [151], and further reduces energy intake by acting on CCK-1 and CCK-2 receptors located along the GIT, vagus nerve, NTS, and hypothalamus [152]. Oxyntomodulin, co-secreted from L-cells, binds the same central GLP-1 receptors, in addition to glucagon receptors in the liver, leading to reduced gastric acid secretion, enhanced satiety, and decreased food intake [148,153]. PYY exists in an inactive PYY1-36 and active dipeptidyl peptidase IV (DPP-IV)-cleaved form, PYY3-36 [153]. PYY3-36, which has high affinity for the Y2 receptor, centrally decreases energy intake by inhibiting orexigenic NPY neurons while activating anorexigenic POMC neurons in the ARC [145,154]. Pancreatic polypeptide (PP) suppresses appetite via Y4 receptors in the AP, NTS, and ARC, and peripherally by reducing GIT motility and secretion [145,155]. Ghrelin, the only major orexigenic gut hormone, is secreted by gastric P/D1 cells during hunger and is also present in the pituitary gland and the ARC (paraventricular nucleus, PVN) [156], where it stimulates NPY/AgRP neurons to promote food intake [157]. Finally, Anandamide, a GIT-produced endocannabinoid, also exerts orexigenic effects by activating cannabinoid receptors 1 and 2 (CB1/CB2) in the CNS and peripheral nervous system, liver, pancreas and adipose tissue [145,158].

### 6.3. Peripheral Mechano- and Chemo-Receptor Brain Interactions

Peripherally, the GIT senses energy and nutrient content via gastric and intestinal mechanoreceptors and intestinal chemo- and nutrient sensors on EECs (Figure 3). Mechanosensitive vagal afferents predominantly innervate the stomach, signalling to the brain in response to gastric distension [159]. This occurs pre-absorption, as this signal relates to volume rather than digested nutrients. Gastric hormones such as ghrelin and leptin reduce the sensitivity of gastric vagal afferent mechanosensors, as shown in ferrets and mice, promoting gastric emptying (a prokinetic effect) and directly stimulating the brain to enhance appetite [160]. Mechanosensitive afferents are also present in the duodenum, which responds to stretch following food consumption and arrival [160]. Chemosensory neurons predominantly innervate the intestinal mucosa and are tuned to detect EEC hormone signals, which in turn, activate cognate receptors at vagal and spinal nerve endings in the lamina propria (local effect), or enter the circulation to act on peripheral and/or central receptors (systemic effect). These actions subsequently convey signals to the brain via vagal and spinal afferents. The gut–brain axis is summarised in Figure 3.

The gut–brain interactions governing nutrient sensing and food consumption described above are complex and remain poorly understood. Further studies to dissect the integrated mechanisms regulating brain responses to nutrient sensing, ingestion and metabolism are crucial for developing effective pharmacological and nutritional strategies to manage obesity and metabolic diseases.

## 7. Conclusions

T2D is a global healthcare crisis, imposing substantial morbidity and mortality, and economic burden. The recognition that nutrient sensing via the gut–brain axis is fundamental to metabolic regulation represents a paradigm shift. Intestinal taste-capability, once viewed as exclusive to the tongue, is now established as a critical nexus in this process, with EECs directly detecting nutrients to regulate eating behaviour, nutrient homeostasis, and metabolic control. This highlights the urgent need to understand how both lingual and intestinal taste signals influence central appetite centres and systemic glucose metabolism to optimise new T2D therapies.

Advancing our knowledge of taste receptor-regulated gut hormones, along with the genetic, environmental, and metabolic drivers governing EEC differentiation, is crucial. Although the current evidence has shown that taste receptors have roles beyond taste sensing and their roles in the control of metabolism and energy intake, some of this knowledge is currently in a preliminary stage, based on indirect evidence or short-term observations. Translating this knowledge to be used in humans can be challenging due to differences in physiology between the species, genetic makeup, varied diet, different doses, follow-up duration, other lifestyle and environmental factors and associated comorbidities. Ultimately, clarifying how taste-capable intestinal EECs orchestrate signalling within the wider enteroendocrine system in humans will provide the potential mechanistic foundation required for developing more targeted and personalised therapeutic strategies for early intervention in metabolic disease.

## 8. Future Directions

Despite the current focus on the cardiometabolic benefits of incretin hormones for treating diabetes and obesity, the mechanisms and environmental factors governing the regulation of taste receptor-equipped EECs and subsequent gut hormone release, along with their central effects, remain largely unmapped.

Active research that further leverages 3D organoid models stands to provide a more physiologically relevant platform for studying these complex interactions [161]. Recent advances demonstrate the utility of organoids, which consist of specific cell types derived from pluripotent stem cells or adult tissue-specific stem cells, and can be grown into human organs in smaller sizes. For example, pancreatic islet 3D models have improved understanding of β-cell function in hyperglycaemic conditions [161], while 3D adipose tissue organoids facilitate the study of adipocyte dysfunction in obesity and T2D [161,162]. Crucially, the capacity to differentiate duodenal and rectal stem cells into mature human EECs using pharmacological agents, such as the CB1 receptor antagonist Rimonabant, ATP-competitive c-Jun N-terminal kinase inhibitor (JNK) SP600125, and Foxo1 inhibitor AS1842856 in ex vivo intestinal organoids [163] offers a novel, controlled environment to interrogate intestinal taste sensing and develop new therapeutic approaches for metabolic disorders. These offer wider revenues for exploring further detailed mechanisms linking the taste receptors and their metabolic and neuronal activities in a human-like environment without posing side effects to participants.

In tandem with these ex vivo and in vivo cellular models, advancements in functional neuroimaging techniques, such as functional magnetic resonance imaging (fMRI), will be essential. fMRI can highlight the dynamic neural pathways, reward centres and central metabolism that link intestinal nutrient sensing and EEC activation to human brain function, taste perception, and food-related behaviour by detecting the changes in blood flow and oxygenation (BOLD signal) of the brain areas that regulate appetite and satiety. fMRI is increasignly used in research for neurological conditions, changes in emotion and reward and psychiatric disorders.

Integrating data from these advanced 3D models and functional neuroimaging represents a promising, multi-faceted approach to fully mapping the gut–brain axis and improving treatment strategies for metabolic disease.

## Figures and Tables

**Table 1:** Principal gut hormones, EECs and their main function in the GIT and appetite regulation in humans [119,120,121,122,123,124,125].

Hormone	Production	Functions
Ghrelin	Gastric PD/D1 cells Pancreatic ε cells	Stimulates food intake, adiposity and gastric emptying
Gastrin	Gastric G cells	Stimulates gastric acid and intrinsic factor secretion from parietal cells; promotes gastric and intestinal motility, mucosal growth
Histamine	Gastric enterochromaffin-like cells	Modulation of GI motility, gastric acid secretion, alteration of mucosal ion secretion
Somatostatin (SST)	Gastric, pancreatic D cells	Reduces gastric acid secretion; limits the release of other gut hormones
Secretin (SCT)	Small intestine S-cells	Stimulate the secretion of pancreatic fluid and bicarbonate
Serotonin (5-HT)	Gastric, intestinal Enterochromaffin cells	Increases motility of the gut
Pancreatic peptide (PP)	Pancreatic PP cells	Inhibits gastric emptying and biliary secretion
Insulin	Pancreatic β-cells	Decreases glucose levels
Glucagon	Pancreatic α-cells	Antagonises insulin effects on hepatocytes, enhances gluconeogenesis and glycogenolysis, promotes oxidation of fat
Amylin	Pancreatic β-cells	Suppresses glucagon secretion, slows gastric emptying, limits food consumption
Glucagon-like-peptide 1 (GLP-1)	Intestinal L-cells	Stimulates insulin; increases beta cell survival, inhibits food intake; reduces gastric emptying and increases satiety
Glucagon-like-peptide 2 (GLP-2)	Intestinal L-cells	Intestinal trophic effect, reduction in gastric emptying
Oxyntomodulin	Intestinal L-cells	Inhibits food intake; reduces gastric emptying
Glucose-dependent insulinotropic polypeptide (GIP)	Intestinal K-cells	Stimulates insulin
Neuropeptide Y (NPY)	GIT enteric neurons	Stimulates food intake
Peptide YY (PYY)	Ileal, colonic L-cells	Inhibits food intake; Reduces gastric emptying
Cholecystokinin (CCK)	Small intestinal I-cells	Inhibits food intake; slows gastric emptying; stimulates pancreatic enzyme secretion and gallbladder contraction
Insulin-like peptide (INSL5)	Colonic L-cells	Enhances appetite
Neurotensin	Ileal N cells	Inhibits postprandial gastric acid secretion and pancreatic exocrine secretion, stimulates colonic motility, inhibits gastric and small intestinal motility

## Data Availability

This was a review article. There is no data collected or available for this manuscript. All information provided in this manuscript was based on evidence derived from the referenced publication.

## Abbreviations

AgRP, agouti-related peptide; ASBT, apical sodium dependent bile acid transporter; AP, area postrema; ARX, aristaless-related homeobox gene; ARC, arcuate nucleus; BTR, bitter taste receptor; CALHM1/3, calcium homeostasis modulator 1 and 3; CaSR, calcium sensing receptors; CB, cannabinoid receptor; CART, cocaine- and amphetamine-regulated transcript; CCK, cholecystokinin; DAG, diacylglycerol; DPP4, Dipeptidyle peptidase-4, EC, enterochromaffin; ECL, entrochromaffin-like; EECs, enteroendocrine cells; ER, endoplasmic reticulum; FLYWCH1, FLYWCH-Type Zinc Finger 1FOXA1, Forkhead Box A1; FOXA2, Forkhead Box A2; FFAR, free fatty acid receptor; fMRI, functional magnetic resonance imaging; GI, gastrointestinal; GIT, gastrointestinal tract; GIP, glucose-dependent insulinotropic polypeptide; GLP-1, glucagon-like peptide-1; GLP-2, glucagon-like peptide-2; GLUT2, glucose transporter 2; GPCR, G protein-coupled receptor; GPR119, G protein-coupled receptor 119; GPBAR1, G protein-coupled bile acid receptor 1; GPRC6A, G protein-coupled receptor family C Group 6 subtype A; HFD, high-fat diet; 5-HT, 5-hydroxytryptamine; IP3, inositol-1-4,5-triophosphate; ISL1, insulin gene enhancer protein; INS5, insulin-like peptide 5; LCFA, long chain fatty acid; MATH1, mouse atonal homolog 1; mGluR, metabotropic glutamate receptor; NEUROD1, neuronal differentiation 1; NEUROG3, neurogenin3; NKX2-2, NK2 homeobox 2-2; NKX6-1, NK6 homeobox 1; NTS, nucleus tractus solitarius; NPY, neuropeptide Y; PAX4, paired box 4; PAX6, paired box 6; PDX1, pancreatic and duodenal homeobox; PP, pancreatic polypeptide; PEPT1, peptide transporter 1; PYY, peptide YY; PLCβ2, phospholipase C-β2; POMC, pro-opiomelanocortin; P2X2/3, purinergic receptors 2 and 3; PROP, 6-n-prophylthiouracil; PTC, phenylthiocarbamide; RPS3, ribosomal protein S3; RUNX1T1, Runt-related transcription factor 1; SCT, secretin; SGLT-1, Sodium/glucose cotransporter 1; SGLT-2, Sodium/Glucose cotransporter-2; B0AT1, sodium-dependent neutral amino acid transporter; SOX4, SRY-related HMG-box 4; STRs, sweet taste receptors; TGR5, takeda G protein-coupled receptor 5; TRC, taste receptor cells; TRIM3, tripartite motif containing 3; TRIM35, tripartite motif containing 35; TRPA1, transient receptor potential cation channel subfamily A member 1; TRPM5, transient receptor potential channel M5; T2D, type 2 diabetes; VGNC, voltage gated sodium channels; ATP, adenosine triphosphate; WT, wild type.
